# A social ecological approach to understanding service utilization barriers among male survivors of sexual violence in three refugee settings: a qualitative exploratory study

**DOI:** 10.1186/s13031-020-00288-8

**Published:** 2020-07-08

**Authors:** Sarah K. Chynoweth, Dale Buscher, Sarah Martin, Anthony B. Zwi

**Affiliations:** 1 Women’s Refugee Commission, 15 West 37th St, New York, NY 10018 USA; 2 Health, Rights and Development (HEARD@UNSW), School of Social Sciences, Faculty of Arts and Social Sciences, The University of New South Wales, Sydney, NSW 2052 Australia; 3 Gender Associations, c/o Ufer Berlin, Paul-Lincke-Ufer 41, 10999 Berlin, Germany

**Keywords:** Sexual violence, Humanitarian aid, Male survivors, Refugees, Service utilization, Social ecological framework, Italy, Bangladesh, Kenya

## Abstract

**Background:**

Post-sexual violence service utilization is often poor in humanitarian settings. Little is known about the service uptake barriers facing male survivors specifically.

**Methods:**

To gain insights into this knowledge gap, we undertook a qualitative exploratory study to better understand the barriers to service utilization among male survivors in three refugee-hosting countries. The study sites and populations included refugees who had travelled the central Mediterranean migration route through Libya living in Rome and Sicily, Italy; Rohingya refugees in Cox’s Bazar, Bangladesh; and refugees from eastern Democratic Republic of the Congo, Somalia, and South Sudan residing in urban areas of Kenya. Methods included document review, 55 semi-structured focus group discussions with 310 refugees, semi-structured key informant interviews with 148 aid workers and human rights experts, and observation of service delivery points. Data were thematically analyzed using NVivo 12.

**Results:**

We identified eleven key barriers and situated them within a social ecological framework to describe impediments at the policy, community (inter-organizational), organizational, interpersonal, and individual levels. Barriers entailed: 1) restrictions to accessing legal protection, 2) legislative barriers such as the criminalization of same-sex sexual relations, 3) few designated entry points, 4) poor or nonexistent referral systems, 5) lack of community awareness-raising and engagement, 6) limited staff capacity, 7) negative provider attitudes and practices, 8) social stigma, 9) limited knowledge (at the individual level), 10) self-stigma, and 11) low formal help-seeking behaviors.

**Conclusion:**

The social ecological framework allowed us to better understand the multifaceted ways that the barriers facing male survivors operate and reinforce one another, and may be useful to inform efforts promoting service uptake. Additional research is warranted in other refugee settings.

## Background

Sexual violence is a significant public health concern. Survivors may be exposed to sexually transmitted infections including HIV, unwanted pregnancy, tetanus, genital and rectal trauma, among other physical impacts [1]. Mental health consequences can include anxiety, depression, post-traumatic stress disorder, and suicidal ideation [1]. Yet service utilization among survivors remains low in many settings, particularly among child survivors [2–4].

Since the early 2000s, gender-based violence and reproductive health actors have made substantial advances in expanding the availability of good quality services for survivors of sexual violence in humanitarian settings. For example, a 2004 global sexual and reproductive health evaluation found a dearth of clinical management of rape in humanitarian settings, whereas such services were more widely available 10 years later [5]. Despite progress, provision of good quality post-sexual violence services remain variable in humanitarian settings [6], due to multiple factors including insufficient funding [7], supply and medicine stock-outs [8], and gender bias within humanitarian agencies [9]. Yet even when post-sexual violence services are available in such settings, service utilization is frequently poor [3, 8]. Research has demonstrated that female survivors face a number of barriers to services including stigma from their community and rejection by their families, which can be aggravated by a lack of provider confidentiality [8, 10]. Less is known about the impediments to service uptake among male survivors in these settings.

From 2018 to 2019, the Women's Refugee Commission's Sexual Violence Project undertook a qualitative exploratory study to better understand the availability and accessibility of post-sexual violence services for male survivors as well as the characteristics and impacts of sexual violence against men and boys. In this paper, we present findings related to the barriers to service uptake among male survivors in Cox’s Bazar, Bangladesh; Rome and Sicily, Italy; and Nairobi and Mombasa, Kenya. We layered these findings within a social ecological framework, which is a theoretical model that helps to identify complex environmental and personal factors that influence individual behavior [11]. The framework includes five levels of influence affecting individual behavior and decision-making: policy, organizational, community (inter-organizational), interpersonal, and individual. By situating the individual within broader social, political, and cultural systems, social ecological models have been historically used to identify factors influencing health outcomes, promote health and social service utilization, and guide public health practice [12]. For this research, the framework was used to organize and synthesize findings, and did not guide the design of the study. Situating the findings within a social ecological framework allowed us to better understand the multifaceted ways that the barriers operate and reinforce one another, and may be useful to inform efforts promoting service uptake.

## Methods

The study explored the characteristics and impacts of sexual violence against refugee men and boys and assessed the availability and accessibility of selected services for male survivors in three refugee settings. Here, we discuss service availability and accessibility; we describe findings related to characteristics and impacts in a separate paper (Chynoweth SK, Buscher D, Martin S, Zwi AB: Characteristics and impacts of sexual violence against men and boys in conflict and displacement: a multi-country exploratory study, submitted). This study was exploratory and qualitative in its approach given the limited research on these issues. Ethics approval was received from the University of New South Wales and the Kenya Medical Research Institute. A global advisory committee comprising 12 experts across a variety of disciplines was also established to provide ethical and technical guidance. In Italy, the University of Palermo’s Department of Psychological, Pedagogical, and Education Services reviewed and provided written approval of the research protocol as official ethics review was not available for non-medical research. In lieu of local ethics approval in Bangladesh, which was cost and time prohibitive, members of our global advisory committee recommended that we establish a national reference group of experienced local practitioners and academics to review the research protocol and tools and provide ethical oversight. National reference groups were also developed for Italy and Kenya to enhance ethical oversight and provide insights into the local context, given the sensitivity and complexity of the issue. The donors for this research had no influence on the study design, data collection and analysis, or drafting of this manuscript.

Study sites were three settings with diverse refugee^1^ populations: Cox’s Bazar, Bangladesh; Rome and Sicily, Italy; and Nairobi and Mombasa, Kenya. Study populations comprised Rohingya refugees from Myanmar living in Bangladesh, refugees and migrants who had travelled the central Mediterranean route living in Italy, and refugees from eastern Democratic Republic of the Congo (DRC), Somalia, and South Sudan residing in urban settings in Kenya. Study site selection criteria included: 1) evidence of widespread conflict-related sexual violence against women and girls in the countries of origin or transit; 2) limited evidence of sexual violence against men and boys in countries of origin, transit, and/or host countries; 3) support by a humanitarian agency to help facilitate in-country data collection. This is not a comparative study; however, geographically varied sites reflecting diverse forms of forced displacement (camp, urban, and peri-urban) were chosen to increase insights into barriers to service availability. The study focuses on persons who were assigned male at birth or who identified as a man or a boy.^2^

We employed four methods to collect data: document review, semi-structured key informant interviews with 148 frontline aid workers (*n* = 137) and human rights experts (*n* = 11; Table 1), 55 semi-structured focus group discussions with 310 refugees (Table 2), and observation of service delivery points. Document review encompassed published research and gray literature, including external and internal UN and international and local non-governmental organization (NGO) documents, focused on sexual violence, gender-based violence, conflict-related human rights violations, and humanitarian service provision for survivors of sexual and gender-based violence in the study sites. Aid workers interviewed included health and mental health providers, gender-based violence specialists, social workers and case managers, and protection and legal aid specialists working with international and national NGOs and UN and government agencies (Table 1). Human rights experts interviewed held expertise in conflict-related sexual violence in the study sites and worked with international and local NGOs and UN agencies.

**Table 1:** Key informants per study setting (*n* = 148)

	Bangladesh	Italy	Kenya	Total
Aid workers working with (*n* = 137):				
Local NGOs	14	32	22	**68**
International NGOs	13	15	12	**40**
UN agencies	13	10	4	**27**
Government agencies	0	2	0	**2**
Human rights experts	5	4	2	**11**
**Total**	**45**	**63**	**40**	**148**

**Table 2:** Refugee focus groups per study setting

	Bangladesh^**a**^	Italy^**b**^	Kenya	Total
Boys (15–17)	3	3	4	**10**
Girls (15–17)	1	0	0	**1**
Young men (18–24)	4	4	4	**12**
Men (24–65)	7	1	4	**12**
Women (24–65)	3	0	4	**7**
Men with physical disabilities (18–65)	3	0	4	**7**
Persons with diverse SOGIESC (18–65)	0	2	4	**6**
**Total**	**21**	**10**	**24**	**55**

^a^ Focus groups with Rohingya with diverse sexual orientation, gender identity and expression, or sex characteristics (SOGIESC) were not convened due to the inability to identify safe spaces in which to hold the discussions at the time of data collection

^b^ Focus groups with women and girls were not convened given the low number of female refugees and migrants entering Italy and the high levels of sexual violence-related trauma within this population. Focus groups with men with disabilities were not convened given the low number of men with disabilities within this refugee and migrant community

Key informants were identified through purposive and chain referral sampling based on their professional role, expertise, and the mandate of their employing agency. Local and international humanitarian agencies engaged community mobilizers to recruit refugees for focus group discussions based on gender identity, gender assignment, sexual orientation, age, and nationality in order to speak with a mix of refugees with diverse life experiences. Community mobilizers emphasized that participation was voluntary and refusal to participate would not result in any repercussions. Monetary or material incentives were not provided to the participants, apart from reimbursement for travel costs (where applicable) and basic refreshments. Informed verbal consent was requested and received from all adult participants; parental or guardian consent was obtained for focus groups with refugees aged 15 to 17, in addition to informed assent.

The data collection tools were originally developed by the principal investigator for a similar study commissioned by the United Nations High Commissioner for Refugees (UNHCR) in 2016 [13]. The tools were refined for this study, specifically the rephrasing and combining of some questions. The key informant interview tool addressed service provision for male survivors, knowledge, attitudes, and behaviors of humanitarian responders with regard to sexual violence against men and boys, and barriers and enablers to accessing services. The focus group discussion tool addressed incidents of sexual violence against men and boys, community knowledge, attitudes, and behaviors related to sexual violence against men and boys, and barriers and enablers to accessing services. In accordance with World Health Organization (WHO) recommendations on researching sexual violence in conflict, we probed only second- and third-hand accounts of sexual violence [14]. However, some refugee research participants spontaneously disclosed their own victimization. To identify indications of distress during an interview or focus group and respond accordingly (including to spontaneous disclosures of victimization), we modified an existing interview distress protocol [15]. Research participants were oriented to and given translated information and consent forms; local service providers offering medical and psychosocial assistance to refugees were listed on the forms.

In-country data collection was undertaken between July 2018 and May 2019. Given the complexity and sensitivity of the subject, the researchers who conceptualized and designed the study undertook the data collection (authors SKC, DB, SM); each have a minimum of 20 years’ experience in undertaking research on sexual violence or other sensitive topics in humanitarian settings. Interviews with key informants each lasted 45 to 60 min and were conducted in English; in Bangladesh and Italy, some interviews were simultaneously translated from Bangla and Italian into English by trained interpreters. Focus group discussions were completed within approximately 1 hour, averaged six participants, and were simultaneously translated from the Rohingya language, Italian, Kiswahili, Lingala, Dinka, and Somali into English by trained interpreters with experience in translation for refugee communities. Field teams were familiar with concerns around the role of interpreters in translating sensitive community issues and carefully selected interpreters who were oriented to and signed a code of conduct committing to confidentiality, neutrality, and respect. Due to the sensitivity of the topic, verbatim electronic notes, rather than audio or video recording, were taken to promote ease among focus group participants. Names of refugee focus group participants were not requested. Services for male survivors assessed included medical, mental health, legal aid, and livelihoods.

**Fig. 1:**
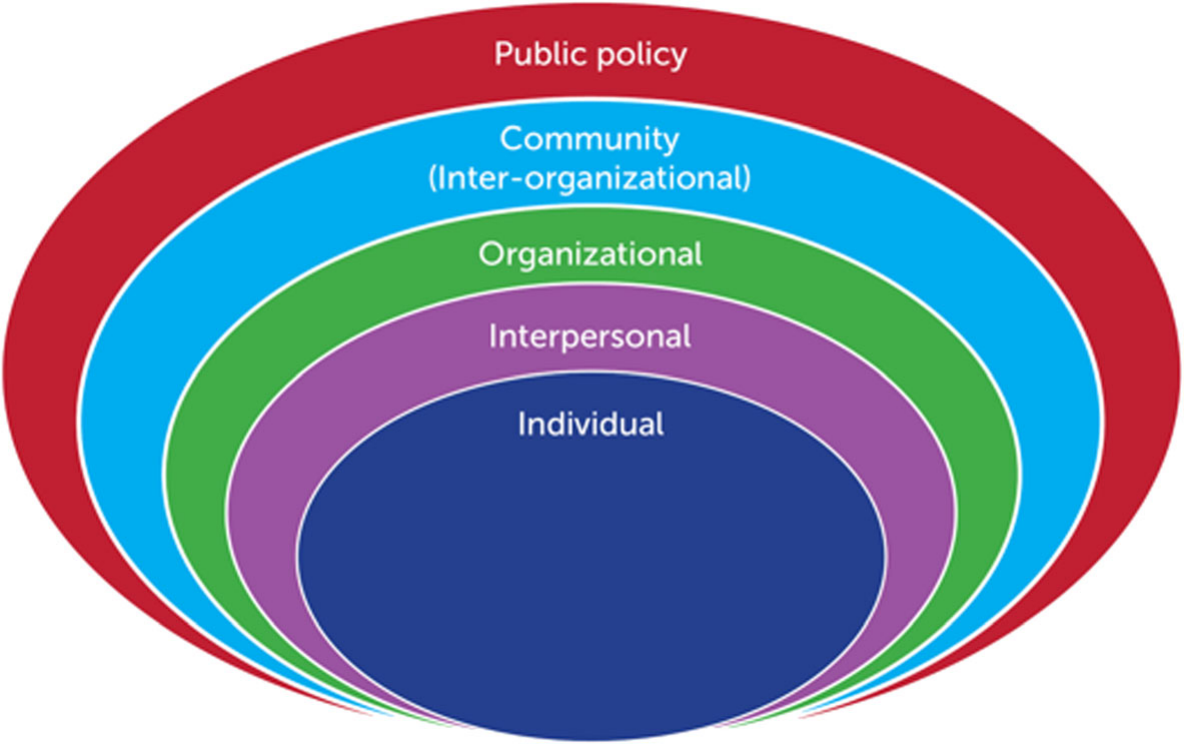
Social ecological framework

The principle investigator undertook multiple waves of coding, and codes were discussed with fellow data collectors to enhance accuracy. Initial coding was undertaken after each field mission. Analysis of the codes revealed themes that aligned with the social ecological framework (Fig. 1), and a final wave of coding was undertaken to situate the findings within the model, which provides a helpful typology for examining, sorting, and presenting insights. Note that the social ecological framework was used during analysis and did not guide data collection; this framework provides useful broad categories for analyzing layers of insights but does not pre-determine the content of such insights. Data were thematically analyzed [16] using QSR International’s NVivo 12, a qualitative data analysis software.

Upon completion of data collection in each study site, preliminary findings were verbally shared and discussed with selected key informants and external experts with expertise in sexual violence in the study sites to verify findings. Follow-up with key informants was undertaken for several months after each data collection mission to clarify inputs, triangulate data, and verify findings. Findings were also triangulated with the results of the document review. Draft written findings were shared with all key informants (*n* = 148), global advisory group members, and national reference group members for review to enhance validity. We subsequently developed and shared translated, simplified summaries of the research findings with operational humanitarian agencies, which distributed these to the refugee communities in the study sites [17]. This formed part of our ethical commitment to provide our findings and plain language analyses back to the communities and organizations that supported and offered insights into our research.

## Results

### Service availability

Availability of specialized services for male refugee survivors of sexual violence was determined through interviews with service provider representatives and triangulated through interviews other key informants and focus groups discussions with refugees. Across all settings, we identified at least one provider offering specialized medical care, legal aid, and mental health and psychosocial support for male refugee survivors (Table 3), although demand outweighed supply, coverage was limited, and quality was variable. Targeted livelihood support for male survivors was probed but was not identified or not available at the time of data collection. More services were identified in Nairobi, particularly for persons with diverse SOGIESC. A number of other agencies, including governmental agencies, offered some components of medical, mental health, and legal aid services where male refugee and migrant survivors may theoretically receive care, but these services were not designed to be inclusive of male survivors and staff were not trained to care for male survivors; these services were omitted from the table below.

**Table 3:** Specialized post-sexual violence services for male survivors per study setting

	Bangladesh	Italy		Kenya	
	Cox’s Bazar	Rome	Sicily	Nairobi	Mombasa
**Health care: clinical care for sexual violence survivors**	-Médecins Sans Frontières (MSF)	-Salute Migranti Forzati (SaMiFo) -Medici Contro la Tortura (MCT)/MSF -Medici per i Diritti Umani (MEDU)	-MEDU	-Kenyatta National Hospital -LVCT Health -MSF -Nairobi Women’s Hospital - National Council of Churches Kenya (NCCK) at Jumuia Huruma Hospital -RefugePoint	-Nairobi Women’s Hospital (Mombasa branch)
**Psychological care: mental health and psychosocial support**	-Action Against Hunger^a^ -BRAC ^a^ -International Organization for Migration^a^ -MSF^a^ -Technical Assistance Inc. (TAI)^a^	-SaMiFo^b^ - MCT/MSF^b^ -MEDU^b^	-Center for Transcultural Psychiatry^b^ -Centro Penc Association^b^ -MEDU^b^	-Center for Victims of Torture^b^ -HIAS^a^ -Health Options for Young Men on HIV/AIDS & STIs (HOYMAS)^a^ -Kenyatta National Hospital^b^ -Kituo Cha Sheria^a^ -LVCT Health^a^ -MSF^b^ -Nairobi Women’s Hospital^b^ -Refugee Consortium of Kenya^a^	-Nairobi Women’s Hospital (Mombasa branch)^b^ -Trace^a^
**Legal aid: to access justice in the refugee host country**	-Bangladesh National Women Lawyers Association -TAI	-Differenze Donna	None identified.	-Kituo Cha Sheria -HIAS -HOYMAS -Refugee Consortium of Kenya -UNHCR	-Kituo Cha Sheria
**Livelihood programs: to support economic wellbeing**	None identified.	None identified.	None identified.	None available at the time of data collection.	None identified.

^a^ These organizations provided psychosocial support, such as psychological first aid, counseling, and support groups

^b^ These organization provided comprehensive mental health care, including psychiatric care

Despite some service availability across settings, numerous barriers impeded male survivors’ service utilization. Barriers were identified at all levels of the social ecological framework (Fig. 1), including policy, community (inter-organizational), organizational, interpersonal, and individual levels. We describe these impediments at each level of the framework.

### Public policy

#### Restrictions to accessing legal protection

In recent years, the governments of the three countries of study have enacted measures restricting asylum-seekers’ access to legal protection, which can deter survivors without some form of formal refugee status from coming forward to access services. For example, the Government of Bangladesh has not granted refugee status to Rohingya who arrived post-August 2017 [18], while the Italian government abolished its “humanitarian protection” residency permit in 2018 and has passed additional restrictions to protection [19]. In Kenya, various laws enacted since 2014, such as a mandatory encampment policy and the revocation of Somalis prima facie refugee status, have severely restricted urban refugees’ access to some form of humanitarian documentation [20]. During focus groups across settings, refugees without formal refugee status (who comprised the large majority of participants) expressed reluctance to making any type of complaint for fear of compromising their ability to access protection or other services/benefits. Others expressed fears of arrest or deportation. An adolescent boy from South Sudan described the difficulty in accessing services without legal protections:

“It is impossible to walk outside today, the UN doesn’t give me a document for Nairobi, I only have for Kakuma. If they see that, [the police] will arrest me… Those who do not have the document cannot even go to the police. Without a document, no one can help you.”

#### Legislative barriers

Restrictive policy frameworks regarding persons with diverse (i.e., non-heteronormative) SOGIESC and narrow definitions of sexual violence further undermined service uptake among male survivors. In Bangladesh and Kenya, same sex sexual relations are criminalized under so-called “unnatural offenses,” with “carnal intercourse” punishable by life imprisonment in Bangladesh [21, 22]. Such legislation deters male rape survivors from seeking services as the physical act itself is criminalized; survivors with diverse SOGIESC are doubly dissuaded. In Kenya, a gender-based violence program officer shared:

“The penal code that outlaws same sex relations--the police and government institutions are using that penal code to really fight the LGBT community… If [a man] reports sexual violence in a government health facility, they probably will not help [him]. ... In fact, you may be in much more trouble if you report—they will say you will be part of the LGBTI group.”

In addition, though Italy and Kenya maintain more inclusive definitions of sexual violence under the law, the Bangladeshi penal code defines rape in terms of male perpetrators and female victims [23].

### Community (inter-organizational)

#### Few designated entry points

Across settings, research participants reported that there were few designated entry points for male survivors to access services. Key informants said that male survivors were reluctant to access care through women-oriented service points, such as post-sexual violence care linked with maternity-related services or women’s centers focused on responding to gender-based violence. At the same time, the absence of entry points forced some male survivors to seek care at women-only spaces, which service providers noted could compromise these spaces for women and girls. In Bangladesh, key informants said that entry points for male survivors were not considered in the response to the 2017 influx, with one gender-based violence specialist noting that the neglect was cross-sectoral: “It is such a failure of the [Humanitarian Response Plan] to not have considered entry points for male survivors in this crisis. Across all sectors... It’s a real failure of this response.”

#### Poor or nonexistent referral systems

Even when male survivors sought care, functioning referral systems for male survivors were not in place in the study sites except among select service providers in Nairobi. In Bangladesh, although humanitarian agencies had developed a gender-based violence referral pathway, service points for male survivors and survivors with diverse SOGIESC were not integrated. A gender-based violence specialist linked the lack of referral points with humanitarian responders’ oversight of male sexual victimization: “Men and boys were not integrated into the referral system because the violence against them was not recognized—by anyone.” In Mombasa, Kenya, few services were in place for male refugee survivors and referral was ad hoc. In Italy, national standard operating procedures on responding to sexual violence had not been operationalized, including the establishment of standardized referral systems. Referral processes were informal, as reflected in the comments of a key informant in Rome: “In the [refugee and migrant] reception system, they are at the mercy of the people in the center. The social workers—how committed are they? What services do they know? Who will they refer to? What connections? How burned out are they?”

### Organizational

#### Lack of community awareness-raising and engagement

Although some services for male survivors were available in the study sites, many providers did not advertise as such. Apart from Nairobi, where a few NGOs were conducting awareness-raising of the available services for female and male refugee survivors, consistent community engagement efforts on post-sexual violence information and services for male survivors were not identified in the study sites. As a result, awareness of the available services was poor: few focus group participants could name one available service for male survivors, even in settings where multiple services and some awareness-raising efforts were in place. A gay Congolese refugee in Nairobi shared, “We are ignorant about the services that are available. We don’t even know where to go if you have an infection.” In Mombasa, refugees repeatedly said that they had “no idea” whether there were any services for male survivors. An adolescent refugee boy in Sicily was unaware of the availability of some local services for boy survivors:

“In Libya, they want to even rape us and have sex with us [the boys]. … They burn you on your private parts. … Why is it only the women that [service providers] talk about with teenage pregnancy, violence against women, and offering programs for them? We don’t understand why no one cares about boys?”

Note that services and programs for women and girls, who bear the brunt of sexual violence according to research participants in all study sites, were also limited across settings.

#### Limited staff capacity

Across settings, key informants reported that service provider capacity to respond to sexual violence survivors--male and female--was generally low, apart from a few exceptional service points. Few providers had received specialized training on caring for male or female survivors, particularly those with diverse SOGIESC, and awareness of male sexual victimization among refugees was generally poor. In Cox’s Bazar, for example, some service providers did not believe that Rohingya men and boys had been subjected to sexual violence. Across settings, service providers failed to recognize and document common forms of sexual violence against men and boys, such as genital violence and forced witnessing. A mental health provider in Bangladesh commented: “We don’t think that genital violence is sexual violence. But it is sexual violence. We don’t know this yet.” A gender-based violence specialist in Bangladesh shared, “We did train…several people on clinical management of rape, but only midwives—the one person a man will never want to see in his life.” A key informant in Sicily described challenges during a training with shelter providers for refugees and migrants:

“It became obvious that the shelter providers have a very limited awareness of the sexual violence against men and boys. We see this and we are trying to adapt prevention materials and awareness-raising techniques in order to open the discussions and explore more and more possibilities to design referral mechanisms for men in the public sector. Even when minimum standards and procedures are in line, the front line workers overlook [male survivors].”

Across settings, key informants reported that health provider capacity to effectively provide clinical management of sexual assault for male and female survivors needed significant strengthening.

#### Negative provider attitudes and practices

Refugees frequently cited negative attitudes by service providers and staff as a key deterrent to service use, including discrimination, disbelief, lack of empathy, and humiliating comments. Research participants reported that some providers may laugh at a male survivor, saying some variation of: “You are a man, you need to defend yourself. How can a man be raped?” During key informant interviews, some service providers expressed problematic misconceptions, for example, that perpetrators of male-on-male sexual violence are gay or that heterosexual male survivors become gay through penile-anal rape. Focus group participants said that racism and xenophobia were particularly harmful and deterred refugees from accessing any kind of services, including post-sexual violence care. Gay and transgender refugees reported that homophobic or transphobic provider attitudes significantly hindered service-seeking behavior, and refugees with disabilities shared that staff may “look down” on them due to their disability, impeding uptake. Some research participants reported that providers may refuse refugee survivors services, due to homophobia or transphobia, racism, or xenophobia. A gay Congolese man living in Nairobi shared: “I went to an NGO when I was raped... I didn’t tell them everything because of fear. They don’t want to know that I’m gay. Once they find this out in the hospital, it’s a big problem. They refuse to treat you.” Refugees across settings were particularly concerned about confidentiality processes, which they cited as the most critical aspect to service uptake. These concerns may be legitimate, as reflected in the comments of a key informant working with gay and transgender refugees in Nairobi, “Even sensitized health care providers, they gossip about them, ‘He has a boyfriend, he should have a girlfriend. Look at this discharge in the rectal area.’ They are gossiped [about] and laughed at by health care providers.”

### Interpersonal

#### Social stigma

Research participants underscored that fears of social stigma and social sanctions by family and community members were significant barriers for male survivors seeking services. Refugees across settings reported that survivors’ primary concern was that their victimization would become known to community and family members, both locally and in their country of origin. They consistently said that male survivors would be shunned, humiliated, and ostracized. Adolescent boy survivors might be blamed for the assault and rejected by their families. The stigma and ostracism can extend not only to the survivor but his family as well. A Somali woman shared: “On behalf of the son [who was victimized], the community will abuse the family. They cannot even live in the community anymore, all the family will be impacted. Because it happened to their son, they will have to leave. Even if he is working with [the community], they will shun him.” Across settings, a number of refugees used the same language, saying that the male survivor is “no longer [seen as] a man.” Gay and transgender refugees were concerned about the exposure of their sexual orientation or gender identity, which could compromise the security and well-being of themselves and their families. An Eritrean man in Italy noted the difficulties of disclosure: “If a man sees that a woman is looking at him [and knows that he was sexually victimized], he’d kill himself. It is terrible for our culture. You would never get to the step of telling about it.” Refugees said that heterosexual male survivors feared being perceived as gay, which can also result in stigma, shame, and social rejection. Male survivors whose victimization could not be attributed to armed groups--such as sexual abuse by a family or community member--reportedly faced additional challenges to disclosure. A Rohingya key informant in Bangladesh commented on sexual abuse of boys within the refugee community: “The biggest barrier is with the family members and the community… There are no ears to hear this. [Boy survivors] are very afraid to share.” A few refugees said that the taboo nature of the topic was protective in nature, as not discussing sexual violence would allow the victim to avoid social repercussions. An older Somali man explained: “There are a lot of men who are victims of abuse, but it is not easy to talk about it in the community. No one is sharing it, so the dignity of that person is preserved.”

### Individual

#### Limited knowledge

Focus group participants (male and female) were largely unaware of the benefits of seeking care, and many did not know that medicine such as post-exposure prophylaxis to minimize HIV transmission existed. One health facility in Nairobi estimated that, due to delays in seeking care, 60% of male survivors who sought services at their clinic were ineligible for post-exposure prophylaxis, which must be taken within 72 h post assault. Some refugees expressed skepticism that services would be helpful or that recovery was possible. Service providers reported that male survivors were often unaware that their mental distress was linked to sexual victimization, and a number of survivors were unaware that other men and boys had been victimized as well. Further, many male survivors reportedly did not realize that the violence they had experienced constituted sexual violence, which may be understood as penile-anal rape only or perpetrated against women and girls alone. Younger boys may not comprehend what constitutes sexual abuse, including appropriate and inappropriate touch, as reflected in the comments by a key informant in Cox’s Bazar: “There are many children and youths and younger children [who have been sexually abused]. They think, ‘What has happened to me? I don’t know what to do.’ They have no knowledge about this [sexual abuse]. That’s why they can’t share.” A health provider working with refugees and migrants who had travelled through Libya commented:

“Electroshock to the genitals is something that they do a lot in Libya. Men don’t know that this is sexual violence because they are being tortured so much in different ways. They don’t know that it’s sexual violence until we give the [awareness-raising] speech. … Most [male refugees and migrants] have no idea about the medical consequences of sexual violence. They lack of knowledge of male sexual violence--people don’t know that we can prevent or treat [the medical impacts].”

The same health provider reported that, as a result of awareness-raising among men and boys on a search and rescue ship in the Mediterranean about the benefits of seeking post-sexual violence medical care, the proportion of survivors accessing medical care who were male increased from 3% in 2017 to 33% in 2018.

#### Self-stigma

Another barrier identified on the individual level was self-stigma, which refers to the internalization of negative public attitudes and beliefs [24]. Research participants underscored that shame and self-blame, often resulting from religious taboos and social constructions of heterosexuality and masculinity, impeded service use. Male survivors sometimes blamed themselves for the assault, particularly those subjected to forced witnessing of sexual violence against women and girls. A Rohingya key informant said: “This is a very horrible moment for us. Men are very angry, very angry. They always say, ‘We are not able to save our girls. And this is our fault.’ They always blame themselves.” A mental health provider in Italy discussed the impacts of rape by guards of a Libyan detention center on a young refugee man:

“He started to think about guilt, sin, and shame. His family was strict Muslim and he couldn’t share it with them. He felt like there is something wrong about him, something in him, that attracted the guard. Another thought is that because he didn’t obey his father and this [rape] was the punishment. He thinks, ‘So maybe if I had done what my father wanted me to do, this wouldn’t [have] happen[ed].’”

Mental health service providers reported that sexual victimization can cause identity-related confusion among heterosexual, cisgender male survivors, who may believe that rape “turned them gay” or that they are “no longer a man.” Some persons with diverse SOGIESC reportedly blamed themselves for the assault, which they perceived as deserved punishment for transgressing sexual and gender norms.

#### Low formal help-seeking behaviors

Although not mentioned by research participants, document review revealed that many refugee populations have low formal help-seeking behaviors [25], including communities who participated in this study such as Somalis [26] and Rohingya [27]. Refugees may prefer to seek help from traditional healers, religious leaders, elders, or community members, particularly for mental health related issues [26, 28]. Mental health service utilization may be particularly challenging to promote as psychological problems remain stigmatized among many refugee communities who, in addition, may not be familiar with western therapeutic models [29]. In focus groups, refugees expressed skepticism and apprehension of service providers generally, including concerns about racism and xenophobia. Wariness of service providers has been documented among refugee populations in other settings [30, 31] as has mistrust of others by communities who have suffered human rights violations such as widespread sexual violence [32].

### Additional barriers

Additional impediments to service utilization were identified in some, but not all, settings. In Italy, communication barriers due to a dearth of cultural mediators and interpreters at service points was often cited as a deterrent to care for all refugees, not only sexual violence survivors. In Kenya, research participants cited financial constraints, including transportation costs, as a key obstacle to service uptake: although free care is available in public facilities, refugees reported that male survivors sometimes prefer private or distant facilities due to confidentiality concerns. Refugees in Kenya also said that fears of retaliation by abusers prevented male survivors from accessing care, and refugees with disabilities reported that limited mobility and a dearth of disability-friendly services hindered service usage broadly.

## Discussion

Using thematic analysis, we identified barriers to service utilization among male refugee survivors of sexual violence and layered them within the social ecological model. To our knowledge, this study is among the first to explore barriers to service uptake among male survivors in refugee settings and is the first to situate findings within a social ecological framework. The framework, which has long been employed to promote health and social service utilization among women, is useful to identify barriers operating in structural, interpersonal, individual, and other ways to influence a survivor’s decision-making regarding care. The framework can help promote a more comprehensive, multi-faceted conceptualization of the barriers that male survivors face, transcending narrow understandings that focus solely on cultural norms or challenges with service provision. Efforts to systematically and sustainably encourage service uptake among male survivors must address the constellation of factors at all levels of the framework, and not simply replicate interventions designed for women and girls.

Many barriers mutually reinforce one another, operating symbiotically to impede service utilization. For example, limited knowledge among service providers of male sexual victimization contributed to the lack of development of entry points and referral processes for male survivors, including those with diverse SOGIESC. The criminalization of same sex sexual activity promotes discriminatory attitudes and practices towards persons with diverse SOGIESC, including among service providers [33].

An important finding was that male survivors frequently did not conceptualize their experiences as sexual violence. Research from the US has found this recognition to be key to service use among heterosexual, gay, and bisexual male survivors [34]. This highlights the importance of awareness-raising and community engagement, with one health provider reporting having observed a direct link between systematic awareness-raising about male sexual victimization and confidential service availability with increased service uptake among male survivors.

Young boys and adolescent boys are particularly vulnerable to sexual violence, abuse, and exploitation in displaced settings [35]. The findings from our study suggest that boys, particularly adolescent boys, in contrast to adult men, face specific or greater barriers to service uptake at different levels of the social ecological framework. They may have less knowledge and understanding of what constitutes abuse, and they may be less aware of available services. Adolescent boys who disclose sexual violence may be blamed for the assault; other research has found that they may be perceived as the perpetrator rather than victim [35]. Boys are frequently abused by someone they know [35], which may be more difficult to disclose than sexual violence by armed groups--a pattern which has been found on research on sexual violence against girls [36, 37]. Boys with disabilities are at greater risk of sexual abuse and less likely to disclose abuse than their non-disabled counterparts [38]. Other research has found that even when boys (and girls) do disclose, service utilization is very low, also due to multiple factors including stigma, fear of repercussions, and lack of information [39], which aligns with our findings.

Persons with diverse SOGIESC are also at heightened risk of sexual violence in conflict and displacement [40, 41] yet the results reveal that they encounter some specific barriers to service usage in contrast to their cisgender, heterosexual counterparts. At the public policy level, the criminalization of same-sex sexual relations is a clear impediment to accessing any sort of services, not only post-sexual violence care. While staff capacity was low for male and female survivors, providers were particularly untrained and unsensitized to care for survivors with diverse SOGIESC. Homophobic and transphobic attitudes and discrimination, and fears thereof, strongly deterred survivors; research in other settings has found that survivors who are male and have diverse SOGIESC are less likely than cisgender, heterosexual survivors to seek care [34] or encounter violence when trying [42]. Without integration into referral systems and targeted awareness-raising to these communities, survivors with diverse SOGIESC will fail to get the care they need and have a right to. This neglect is systemic in the humanitarian system: none of the largest Humanitarian Response Plans in 2018 addressed persons with diverse SOGIESC [43].

It is critical to recognize that many of the identified barriers apply to female survivors as well, such as restricted access to legal protection, limited provider capacity, and negative provider attitudes. Women and girls may face additional or unique barriers to reporting, such as practices that force female survivors to marry their rapists and child-care responsibilities that prevent women from seeking care [10]. Indeed, the failure to provide accessible services to male survivors may compromise female survivors’ ability to access care, as reflected in the example of the lack of entry points spurring men and boys to approach women-friendly spaces, which can dissuade women and girls from accessing these services.

Although this study spotlights the specific barriers facing male survivors, comprehensive efforts should be undertaken to address barriers and promote post-sexual violence service utilization among each and every survivor, including adult women and men, children and adolescents, the elderly, gay and bisexual men, lesbian and bisexual women, trans men and trans women, and persons who do not conform to the gender binary. Efforts addressing negative provider attitudes and practices, for example, should be inclusive and address a range of myths and misconceptions, such as the myth that female survivors “attract” an assault through “provocative” dress and male victims who experienced an erection during rape—a common physiological response—must have “enjoyed” it. A survivor -centered approach based on the gender-based violence guiding principles of safety, respect, confidentiality, and non-discrimination should underpin efforts to develop good quality care for survivors of all genders.

The specific barriers identified largely align with other studies that included documentation of barriers to service accessibility among male survivors in refugee settings [13, 44]. Additional barriers emerged in other refugee settings, such as limited hours of operation that prohibited refugees who worked from accessing care [13], insecurity [44], the public nature of pursuing legal redress [13], and national laws stipulating the mandatory reporting of public officials [13]. More research on the specific service utilization barriers facing men, adolescents, and boy survivors, including those with physical, psychological, and intellectual disabilities, is warranted in other settings of forced displacement as well as a disaggregated approach to the specific barriers of persons with diverse SOGIESC, including gay men, trans women, trans men, lesbians, nonbinary persons, and intersex persons. Impediments experienced by persons with multiple intersecting vulnerabilities, such gay men or boys with disabilities, merits further research as well.

## Limitations

This study faced limitations. We were not able to meet the requirements of some best practices in qualitative research, such as the use of multiple independent coders, due to time and resource constraints. However, where possible, we undertook additional measures to enhance the quality of data collection and analysis, such as the establishment of global and national advisory committees, sharing the draft findings with all 148 key informants as well as external experts for review and feedback, and long-term follow-up with key informants to clarify ambiguities and verify findings. Sexual violence against men and boys is a complex and sensitive issue for all study populations and, at times, the issue was difficult to discuss with refugee focus group participants; we underscored informed consent and emphasized participants’ right to skip questions or leave, without repercussions. Translation error is a possibility, particularly in Bangladesh, as Rohingya interpreters with training in gender-based violence were rare and refugee focus group discussions were held in Chittagonian, a similar but not identical language to the Rohingya language. Given the taboo nature of the subject and the potential for harm resulting from confidentiality breaches or insensitivity, we decided to prioritize engaging Chittagonian-speaking interpreters with expertise in gender-based violence rather than Rohingya-speaking interpreters. The composition of the data collection teams may have directly or indirectly influenced focus group dynamics. The data collectors were Americans and Europeans and the interpreters were members of the refugee and/or host communities. The “outsider” status of the data collectors and the “insider” status of the refugee interpreters may have influenced refugee research participants to disclose or withhold information. We were unable to undertake focus group discussions with Rohingya with diverse SOGIESC in Bangladesh and trans men across all settings due to the inability to safely access these communities. To address this, we collected data on these groups through service providers. Note-taking error is also a possibility. Utilizing focus groups as a method may produce more socially accepted narratives, such as focusing on sexual violence perpetrated by armed groups as opposed to sexual abuse by family or community members, which may be more difficult to openly speak about [36]; conducting individual interviews may have provided further opportunities for exploring sensitive issues in depth. However, in the context of researching sexual violence among conflict-affected communities, WHO recommends individual interviews as a last resort [14], given the vulnerability of the participants and the potential for adverse impacts. The authors and the advisory group members deemed individual interviews with refugees ethically questionable for this study and found that focus groups were sufficient to achieve the research aims. Some biases may have been introduced by purposive sampling, and we may have overlooked some non-traditional actors providing services for male survivors. In Kenya, four focus groups with Somali refugees were held during the first day of Ramadan, during which discussion of negative topics are discouraged, which may have limited discussion of this sensitive topic.

## Conclusions

Multi-dimensional barriers impede service uptake among male survivors in refugee settings. Using a social ecological framework helped contextualize and better understand these barriers at the policy, organizational, community, interpersonal, and individual levels. This, in turn, can help inform holistic interventions to promote service utilization. Additional research on barriers to service uptake among male survivors, including those with diverse SOGIESC, living in settings of forced displacement is warranted.

## Data Availability

The datasets generated during and/or analyzed during the present research are not publicly available to protect the anonymity of participants. Reports of the results from each study site can be obtained from: https://www.womensrefugeecommission.org/svproject.

## Abbreviations

DRC: The Democratic Republic of the Congo
HOYMAS: Health Options for Young Men on HIV/AIDS & STIs
MCT: Medici Contro la Tortura
MEDU: Medici per i Diritti Umani
MSF: Médecins Sans Frontières
NCCK: National Council of Churches Kenya
NGO: Nongovernmental organization
SaMiFo: Salute Migranti Forzati
SOGIESC: Sexual orientation, gender identity and expression, or sex characteristics
TAI: Technical Assistance Inc.
UNHCR: United Nations High Commissioner for Refugees

## Footnotes

1 Few research participants had received formal refugee status; however, in this paper, we use the term “refugee” for ease of reading and because many meet the definition of a refugee as defined in the 1951 UN Refugee Convention and 1967 Protocol.

2 We use the term “men and boys” for ease of reading and acknowledge that it does not capture many persons of diverse sexual orientation and gender identity and/or expression who are included in the scope of the study.

## References

[1] World Health Organization (2003). Guidelines for medico-legal care for victims of sexual violence.

[2] Sumner SA, Mercy JA, Saul J, Motsa-Nzuza N, Kwesigabo G, Buluma R, Marcelin LH, Lina H, Shawa M, Moloney-Kitts M, Kilbane T (2015). Prevalence of sexual violence against children and use of social services—seven countries, 2007–2013. MMWR Morb Mortal Wkly Rep.

[3] Whitmill J, Blanton C, Doraiswamy S, Cornier N, Schilperood M, Spiegel P, Tomczyk B (2016). Retrospective analysis of reproductive health indicators in the United Nations high commissioner for refugees post-emergency camps 2007–2013. Conf Health.

[4] Darnell D, Peterson R, Berliner L, Stewart T, Russo J, Whiteside L, Zatzick D (2015). Factors associated with follow-up attendance among rape victims seen in acute medical care. Psychiatry.

[5] Chynoweth SK (2015). Advancing reproductive health on the humanitarian agenda: the 2012-2014 global review. Conf Health.

[6] Reis C, Ratnayake R. Taking stock of reproductive health in humanitarian settings: 2012–2014 Inter-agency Working Group on Reproductive Health in Crises' global review. Conf Health. 2015;9(Suppl 1).

[7] International Rescue Committee, Voice (2019). Where is the money? How the humanitarian system is failing in its commitments to end violence against women and girls.

[8] Casey SE, Chynoweth SK, Cornier N, Gallagher MC, Wheeler EE (2015). Progress and gaps in reproductive health services in three humanitarian settings: mixed methods case studies. Conf Health.

[9] Reis C (2018). Clinical management of rape in conflict: exploring the persistence of sub-standard humanitarian responses [dissertation].

[10] Wirtz AL, Glass N, Pham K, Aberra A, Rubenstein LS, Singh S, Vu A (2013). Development of a screening tool to identify female survivors of gender-based violence in a humanitarian setting: qualitative evidence from research among refugees in Ethiopia. Conf Health.

[11] Sallis JF, Owen N, Fisher E, Glanz K, Rimer BK, Viswanath K (2008). Ecological models of health behavior. Health behavior and health education.

[12] Golden SD, Earp JA (2012). Social ecological approaches to individuals and their contexts: twenty years of health education & behavior health promotion interventions. Health Educ Behav.

[13] Chynoweth SK. “We keep it in our heart”: sexual violence against men and boys in the Syria crisis: UNHCR; 2017. https://www.refworld.org/docid/5a128e814.html. Accessed 13 Jan 2020.

[14] World Health Organization. Ethical and safety recommendations for researching, documenting and monitoring sexual violence in emergencies. Geneva: WHO; 2007.

[15] Draucker CB, Martsolf DS, Poole C (2009). Developing distress protocols for research on sensitive topics. Arch Psychiatr Nurs.

[16] Guest G, MacQueen KM, Namey EE (2011). Applied thematic analysis.

[17] Women's Refugee Commission. Summary for Community Contributors. 2019. https://www.womensrefugeecommission.org/svproject. Accessed 30 Jun 2020.

[18] Bhatia A, Mahmud A, Fuller A, Shin R, Rahman A, Shatil T, Sultana M, Morshed KM, Leaning J, Balsari S (2018). The Rohingya in Cox’s bazar: when the stateless seek refuge. Health Hum Rights.

[19] Corsi C. Evaluating the ‘Salvini decree’: doubts of constitutional legitimacy. Migration Policy Center. 2019. https://cadmus.eui.eu/bitstream/handle/1814/61784/PB_2019_06_MPC.pdf. Accessed 12 May 2020.

[20] Norwegian Refugee Council, International Human Rights Clinic at Harvard Law School (2017). Recognising Nairobi’s refugees: the challenges and significance of documentation proving identity and status.

[21] Kenya Penal Code, 1930, Sections 162, 165.

[22] Bangladesh Penal Code, 1860, Act No. 45, c. 16, s. 377.

[23] Bangladesh Penal Code, 1860, Act No. 45, c. 16, s. 375.

[24] Deitz MF, Williams SL, Rife SC, Cantrell P (2015). Examining cultural, social, and self-related aspects of stigma in relation to sexual assault and trauma symptoms. Violence Against Women.

[25] De Anstiss H, Ziaian T, Procter N, Warland J, Baghurst P (2009). Help-seeking for mental health problems in young refugees: a review of the literature with implications for policy, practice, and research. Transcult Psychiatry.

[26] Markova V, Sandal GM (2016). Lay explanatory models of depression and preferred coping strategies among Somali refugees in Norway: a mixed-method study. Front Psychol.

[27] UNHCR (2018). Culture, context and mental health of Rohingya refugees.

[28] Adaku A, Okello J, Lowry B, Kane JC, Alderman S, Musisi S, Tol WA (2016). Mental health and psychosocial support for south Sudanese refugees in northern Uganda: a needs and resource assessment. Confl Health.

[29] Byrow Y, Pajak R, McMahon T, Rajouria A, Nickerson A (2019). Barriers to mental health help-seeking amongst refugee men. Int J Environ Res Public Health.

[30] Ní RM (2013). The causes of mistrust amongst asylum seekers and refugees: insights from research with unaccompanied asylum-seeking minors living in the Republic of Ireland. J Refug Stud.

[31] Asgary R, Segar N (2011). Barriers to health care access among refugee asylum seekers. J Health Care Poor Underserved.

[32] Nickerson A, Bryant RA, Rosebrock L, Litz BT (2014). The mechanisms of psychosocial injury following human rights violations, mass trauma, and torture. Clin Psychol (New York).

[33] Clark F (2014). Discrimination against LGBT people triggers health concerns. Lancet.

[34] Donne MD, DeLuca J, Pleskach P, Bromson C, Mosley MP, Perez ET, Mathews SG, Stephenson R, Frye V (2018). Barriers to and facilitators of help-seeking behavior among men who experience sexual violence. Am J Mens Health.

[35] Family for Every Child (2018). Caring for boys affected by sexual violence.

[36] Stark L, Sommer M, Davis K, Asghar K, Baysa AA, Abdela G, Tanner S, Falb K. Disclosure bias for group versus individual reporting of violence amongst conflict-affected adolescent girls in DRC and Ethiopia. PLoS One. 2017;12(4):e0174741. 10.1371/journal.pone.0174741 PMC5380345 28376108

[37] Murphy M, Ellsberg M, Contreras-Urbina M. Nowhere to go: disclosure and help-seeking behaviors for survivors of violence against women and girls in South Sudan. Confl Health. 2020;14(6). 10.1186/s13031-020-0257-2. 10.1186/s13031-020-0257-2 PMC7017609 32082415

[38] Hershkowitz I, Lamb ME, Horowitz D (2007). Victimization of children with disabilities. Am J Orthop.

[39] Ligiero D, Hart C, Fulu E, Thomas A, Radford L (2019). What works to prevent sexual violence against children: evidence review. Together for Girls.

[40] HIAS (2014). Triple jeopardy: protecting at-risk refugee survivors of sexual and gender-based violence.

[41] Rosenberg J. Mean streets: identifying and responding to urban refugees’ risks of gender-based violence. Womens Refugee Commission. 2016. https://www.womensrefugeecommission.org/gbv/resources/1272-mean-streets. Accessed 13 Jan 2020.

[42] Rosenberg J (2016). “Like a stray dog on the street”: trans* refugees encounter further violence in the cities where they flee. LGBTQ Policy J.

[43] Humanitarian Advisory Group (2019). Taking sexual and gender minorities out of the too-hard basket.

[44] Kiss L, Quinlan-Davidson M, Pasquero L, Tejero PO, Hogg C, Theis J, Park A, Zimmerman C, Hossain M (2020). Male and LGBT survivors of sexual violence in conflict situations: a realist review of health interventions in low-and middle-income countries. Confl Health.

